# Evaluation of t-DARPP Expression Alteration in Association with DDR1 Expression in Non-Small Cell Lung Cancer

**DOI:** 10.61186/ibj.3878

**Published:** 2023-06-24

**Authors:** Zahra Damavandi, Pardis Riahi, Tayebeh Majidizadeh, Massoud Houshmand

**Affiliations:** Department of Medical Biotechnology, National Institute of Genetic Engineering and Biotechnology, Tehran, Iran

**Keywords:** Collagen type I, Discoidin domain receptor 1, Non-small cell lung cancer, Phosphoprotein phosphatase-1 regulatory subunit 1B

## Abstract

**Background:**

DDR1 signaling plays a critical role in various cellular functions. Increased DDR1 expression has been shown in different human cancers. t-DARPP is a truncated isoform of DARPP-32, and its upregulation promotes cell survival and migration. Most lung cancer patients have NSCLC, and their survival rate is low. Therefore, it is necessary to study new and effective targeted therapies. Increased t-DARPP expression in NSCLC patients is associated with patient survival and can act as a prognostic marker correlated with increasing stages of NSCLC. The current study aimed to evaluate alteration in DDR1 expression and its effects on t-DARPP expression in NSCLC.

**Methods:**

Two human lung adenocarcinoma cell lines, A549 and Calu-3, were treated with collagen type I and transfected with DDR1 siRNA. The relative expression of DDR1 and t-DARPP was evaluated using qRT-PCR.

**Results:**

The results indicated that collagen type I could stimulate DDR1 expression in NSCLC cells. Also, DDR1 upregulation resulted in a significant increase in t-DARPP expression. In contrast, suppression of DDR1 expression significantly decreased t-DARPP expression.

**Conclusion:**

Our findings propose that modification in the expression of DDR1, caused by collagen type I and siRNA, might influence the expression of t-DARPP in NSCLC that is linked to NSCLC progression. Moreover, this alteration could potentially serve as an innovative target for therapeutic intervention.

## INTRODUCTION

Lung cancer is responsible for the most cancer-related death worldwide, and its main type is NSCLC^[^^1^^]^. Despite numerous progression in oncology, lung cancer treatment has not fundamentally improved. Understanding the pathways involved in the etiology of lung cancer is required to identify critical biomolecules and discover effective targeted therapies^[^^2^^]^. 

DDRs are kinds of tyrosine kinase receptors that recognize specific amino acid motifs in collagen (binding motif: GVMGFO) for binding and activation and comprise DDR1 and DDR2. Moreover, DDRs are important transmembrane regulators that control signaling and cell-matrix interactions^[^^3^^-^^6^^]^. DDR1 is widely distributed in the body but mainly expressed in epithelial cells and activated by fibrillar and non-fibrillar collagens. DDR1 signaling plays a critical role in various cellular functions, including proliferation, extracellular matrix remodeling, survival, migration, and immune response. Increased DDR1 expression in several human cancers, such as renal clear cell carcinoma, breast cancer, NSCLC, prostate cancer, esophageal cancer, and hepatocellular carcinoma, indicates DDR1 impact on oncogenesis^[^^6^^-^^11^^]^. Following the activation of DDR1 tyrosine kinase function, several cytoplasmic signaling molecules, such as DARPP-32, ShcA, Nck2, FRS-1, and SHP-2, bind to DDR1 in a phosphotyrosine system and regulate cell migration and adhesion^[^^12^^,^^13^^]^.

In 1983, DARPP-32 was first described in brain regions enriched with dopaminergic nerve terminals. DARPP-32 is a key signaling regulator molecule activated by a range of neurotransmitters, including glutamate, dopamine, GABA, and serotonin^[^^12^^,^^14^^,^^15^^]^. DARPP-32 is encoded by the *PPP1R1B* gene and named phosphoprotein PPP1R1B, since it acts as an inhibitor of PP-1. Phosphorylation of Thr34 residue on DARPP-32 by PKA causes the inhibition of PP-1. On the contrary, phosphorylation at threonine-34 (Thr75) position by cyclin-dependent kinase-5 inhibits PKA and prevents PKA from phosphorylating DARPP-32. The N-terminally truncated isoform of DARPP-32, called t-DARPP, was found to utilize a unique alternative first exon located within intron 1 of DARPP-32. The t-DARPP lacking 36 amino acids at N-terminal was initially recognized in gastric cancer. Therefore, it does not have a Thr-34 phosphorylation site and does not show PP1 inhibitory activity^[^^16^^,^^17^^]^. Many studies have reported that the aberrant expression of DARPP-32 and t-DARPP stimulates oncogenesis in the adenocarcinomas of the breast, lung, gastric, colon, prostate, and esophagus by regulating survival, proliferation, migration, angiogenesis, and invasion^[^^18^^-^^25^^]^. The t-DARPP overexpression has been shown in several adenocarcinomas and primes cancer cells for their ability to resist apoptosis in a p53-independent manner. Moreover, t-DARPP/Akt axis signaling emphasizes the new carcinogenic role of t-DARPP in cancer cell survival and resistance to drug-induced apoptosis. Studies have indicated the ability of DARPP-32 to suppress the cell growth and the inverse ability of t-DARPP to induce PKA activation and trastuzumab resistance^[^^17^^-^^27^^]^. Also, DDR1 activation has been shown to induce PI3 kinase/Akt and NFκB pathways^[^^28^^]^. It has demonstrated that DARPP-32 and its splice variant t-DARPP stimulate lung cancer cell survival and migration. Moreover, the elevated t-DARPP isoform levels in NSCLC patients are associated with increased tumor staging and poor patient survival^[^^29^^]^. DARPP-32 has been identified as a new DDR1 binding partner in human breast cells and supports the critical role of the DDR1/DARPP-32 complex in motility^[^^12^^]^. Therefore, it seems that DDR1 can influence DARPP-32 isoforms in cancers. However, the t-DARPP signaling and its biological function in oncogenesis has largely been remained unknown. Thus, new studies on the t-DARPP molecular mechanism are highly required. In this study, we analyzed DDR1 expression stimulated with collagen type I and inhibited by siRNA in NSCLC cells. Then we evaluated t-DARPP expression to find correlation of t-DARPP expression alteration with DDR1 expression in NSCLC.

## MATERIALS AND METHODS

**Cell culture**

Human adenocarcinoma cell lines, A549 and Calu-3, were purchased from the National Cell Bank of Iran (Pasteur Institute of Iran, Tehran) and cultured in DMEM with a high concentration of glucose supplemented with 10% FBS (Gibco, USA), 100 U/ml of penicillin, and 100 µg/ml of streptomycin in humidified air with 5% CO_2_ at 37 °C.

**DDR1 stimulation with collagen type I**

Lyophilized rat tail Collagen Ι (Roche, Germany) was dissolved in sterile 0.2% acetic acid (v/v) according to the manufacturer's instructions. Sufficient diluted collagen was added to the surface of coated dishes (5-10 µg/cm^2^) and carefully spread on the bottom of the culture dishes for being coated with collagen type I. Then the plates were incubated in the laminar flow hood at +15 to +25 °C for about 60 min. The coated surface was washed with a medium or PBS buffer. Finally, the cells were implanted in collagen Ι-coated plates in various time points (2, 4, 8, 12 and 16 hours) for DDR1 stimulation with collagen type I.

**Knockdown of DDR1 by siRNA transfection**

When the cells reached 60-70% confluence in each well of six-well plates, transfection of the si-DDR1, a mixture of four siRNA (SMARTpool ON-TARGETplus siRNA, Dharmacon, USA) and control siRNA (ON-TARGETplus non-targeting control siRNA, Dharmacon) into the cells were performed using DharmaFECT Transfection Reagent (Dharmacon) according to the manufacturer’s protocol using 25 nM of final concentration of siRNAs. The cells were grown in a serum-free medium for 6 h. Then 10% FBS was added to the medium, and the cells were again grown for 42 h. Negative control was used to determine the level of transfection efficiency. For this purpose, we evaluated the normalized DDR1 expression with GAPDH. Accordingly, the cells with negative control transfection had 100% DDR1 expression, and the other cells transfected with si-DDR1 had passive (X) percentage of DDR1 expression. 

**RNA extraction and qRT-PCR**

Total RNA was isolated from the cells by TRIzol reagent (Invitrogen, Carlsbad, CA, USA) according to the manufacturer's instructions. The concentration of RNA and A260/280 ratio were evaluated using a NanoDrop Spectrophotometer (Thermo Fisher Scientific, Waltham, USA). RNA quality was determined via agarose gel electrophoresis. Total RNA (1 µg) was reverse transcribed using random primers and RevertUP™ II Reverse Transcriptase (Biotechrabbit™ cDNA Synthesis Kit, Hennigsdorf, Germany) according to the manufacturer’s protocol. The qRT-PCR was performed using the SYBR Green assay in the Corbett Rotor-Gene 6000 Real-time PCR Machine (Qiagen, Hilden, Germany) for evaluating DDR1, t-DARPP, and GAPDH mRNA expression. PCR amplification was carried out using the following conditions: 95 °C for 5 minutes, followed by 40 cycles of 95 °C for 30 seconds, DDR1 and t-DARPP: 59 °C; GAPDH: 61.5 °C for 30 seconds and 72 °C for 30 seconds with a final extension at 72 °C for 5 min. The comparative threshold cycle method (2^-ΔΔCt^) was used to quantify the relative amounts of transcripts with GAPDH as an internal control. Reactions were performed in duplicate, and the results of three independent experiments were subjected to statistical analysis. The authenticity of the amplified products was evaluated by agarose gel electrophoresis.

**Western blotting**

The cells were implanted on 10-cm plates coated with 1 mg/ml of collagen and fibronectin (control). After acceptable confluency, the cells were then lysed using Pierce RIPA buffer (Thermo Fisher Scientific). Protein yields were assigned by BCA Protein assay kit (Thermo Fisher Scientific), and 40 µg of protein was loaded with 1× SDS sample buffer onto 10% or 8% polyacrylamide gels. Gels were transferred onto 0.45-µm nitrocellulose membranes (Bio-Rad, USA) and blocked in 5% BSA. Next, the membranes were incubated overnight with primary goat anti-DDR1 antibodies (1:1,000 dilution; AF2396, R&D Systems, Minneapolis, USA) or mouse anti-β-actin antibodies (1:5,000 dilution; MABT825, MilliporeSigma, Burlington, USA) in 2.5% BSA in TBS containing 0.1% Tween-20 at 4 °C. The membranes were incubated with respective secondary antibody in TBS comprising 0.1% Tween-20 at room temperature for 1 h. Finally, Image Studio (Mandel) was used for detection and analysis of immunoblots. 

**Statistical analysis**

Data were illustrated as mean ± standard deviation with at least three independent experiments. Statistical analysis was performed using GraphPad Prism 8.0 statistical software (GraphPad Software Inc., USA). The mRNA expression levels were normalized to those of GAPDH. The significance of differences among groups was assessed using the Student’s t-test and analysis of variance (ANOVA). The *p* values less than 0.05 were considered statistically significant. 

## RESULTS

**Fig. 1.**
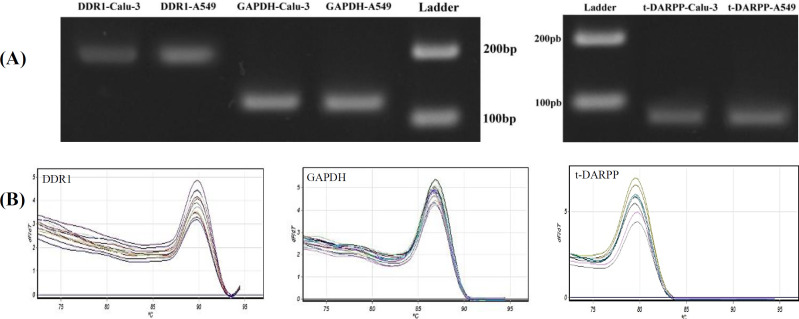
Uniqueness and authenticity of the RT-PCR products. (A) The size and uniqueness of DDR1, t-DARPP, and GAPDH PCR products following agarose gel electrophoresis. (B) The authenticity of the PCR amplified products shown by single, sharp melting curves

We used a qRT-PCR approach with specific primers to amplify *DDR1* and *t-DARPP* genes, which are important for lung cancer treatment. The uniqueness and authenticity of the amplified products were confirmed by agarose gel electrophoresis and the presence of single, sharp melting curves (Fig. 1). The cells were transfected and treated with the shRNA or control complexes in a 96-well plate. After 48 hours, the medium was replaced with 100 μL of a fresh medium. Then 10 μL of MTT solution (5 mg of MTT powder in 1 mL of PBS buffer) was added to each well and further incubated for 4 hours. The formazan crystals formed in viable cells were dissolved in dimethyl sulfoxide. The plate was covered with aluminum foil and mixed at 100 rpm for 15 minutes. Finally, the absorbance was measured at 570 nm using an Epoch microplate reader (BioTek, USA). The experiment was performed in triplicate.

**Results of collagen type I induction and DDR1 knockdown in A549 and Calu-3 cells**

**Fig. 2:**
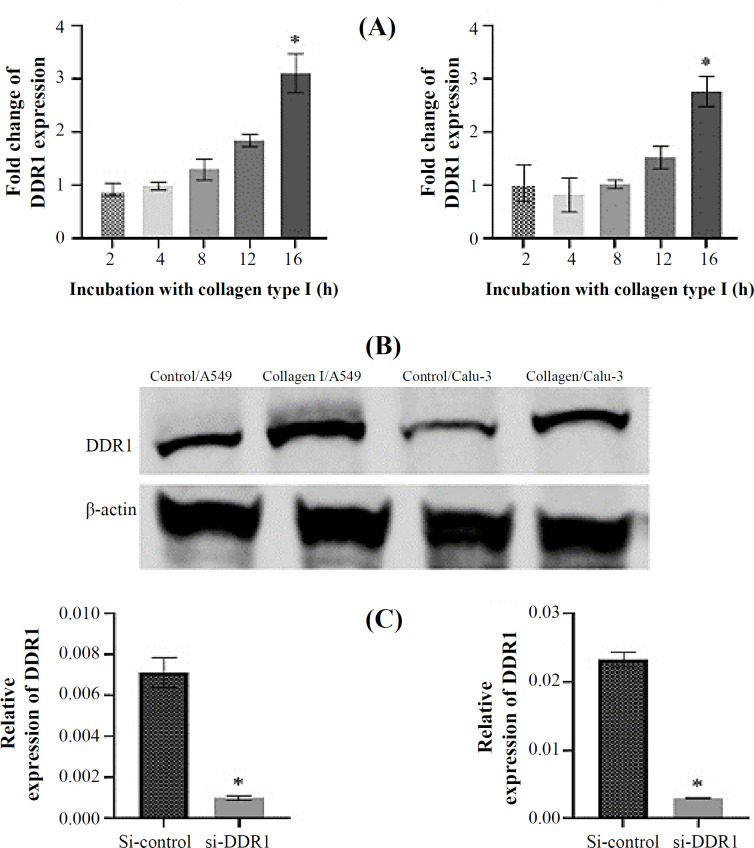
Expression of DDR1 after collagen type I simulation and siRNA transfection in A549 and Calu-3 cells. (A) Expression analysis of DDR1 mRNA in the cells incubated with collagen type I at various times by RT-PCR. DDR1 mRNA expressions increased significantly after 16 hours. The mean fold change of DDR1 expression was calculated relative to unstimulated controls. (B) Western blot analysis of DDR1 protein expression in A549 and Calu-3 cells incubated with collagen type I and fibronectin as control after 16 h; DDR1 expression enhanced under the collagen type I stimulation. (C) Analysis of DDR1 expression after siRNA transfection in cells using RT-PCR. DDR1 mRNA expressions decreased significantly after si-DDR1 transfection compared to non-targeting control siRNA. All experiments were conducted in three biological and two technical replicates (^*^p < 0.05)

A549 and Calu-3 cells were cultured on plates coated with collagen type I and also on the control plates for various times (2-16 h). Total RNA was extracted to perform qRT-PCR to determine whether collagen Ι could promote the DDR1 expression in NSCLC cells. The results showed that the mRNA expression of DDR1 significantly increased in the cells treated with collagen type I compared to the control (Fig. 2A). Also, the total protein expression of DDR1 was detected using Western blotting assay (Fig. 2B). Our data revealed that mRNA and protein expressions of DDR1 were induced in a time-dependent mode; i.e., the high expression achieved after 16 h of collagen type I stimulation. Moreover, the cells were transfected with SMARTpool DDR1 siRNA, a mixture of four siRNAs provided as a single reagent to reduce the expression of DDR1. The level of si-DDR1 transfection efficiency in A549 and Calu-3 cells was 85.7% and 86.9%, respectively. The DDR1 mRNA expression levels significantly decreased in A549 and Calu-3 cells after being treated with si-DDR1 compared to non-targeting control siRNA transfected cells (Fig. 2C). 

**Alterations of** **t-DARPP** **expression in A549 and Calu-3 cells**

**Fig. 3:**
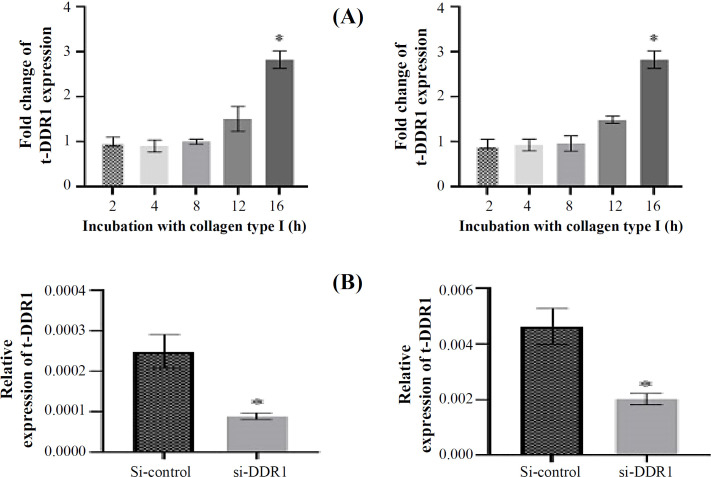
Analysis of t-DARPP expression in A549 and Calu-3 cells after induction by collagen type I and knockdown DDR1. RT-PCR was conducted to evaluate the t-DARPP expression in NSCLC cells stimulated by collagen type I in a time-dependent manner after DDR1 siRNA transfection. (A) Relative t-DARPP mRNA expressions significantly increased in the cells after 16 hours. The mean fold change of t-DARPP expression was calculated relative to unstimulated controls. (B) Relative t-DARPP mRNA expressions significantly decreased after si-DDR1 transfection versus non-targeting control siRNA in cells. All experiments were conducted in three biological and two technical replicates (^*^p < 0.05)

We transiently overexpressed DDR1 with collagen type I stimulation at various time points and knockdowned DDR1 by SMARTpool DDR1 siRNA sequences to study the relationship between the expression of DDR1 and t-DARPP in two NSCLC cell lines (A549 and Calu-3). The qRT-PCR results showed that the mRNA expression level of DDR1 significantly increased after 16 hours. Furthermore, DDR1 mRNA expressions significantly decreased after siRNA transfection. Therefore, we evaluated the expression levels of t-DARPP in the cells after the stimulation of collagen type I and siRNA transfection to confirm the effects of DDR1 on t-DARPP expression. According to the results, t-DARPP mRNA expression level significantly increased after DDR1 overexpression in a time-dependent manner. Furthermore, it was observed that the highest expression of t-DARPP mRNA was attained after 16 hours of collagen type I stimulation (Fig. 3A). Moreover, in both cell lines tested, the knockdown of DDR1 affected the mRNA level of t-DARPP and significantly decreased t-DARPP expressions (Fig. 3B). Our results demonstrated that the inhibition of DDR1 downregulated t-DARPP, whereas the stimulation of DDR1 upregulated t-DARPP. 

## DISCUSSION

NSCLC with a high incidence and low five-year survival rate has one of the worst prognoses among cancers. Hence, it is required to discover effective targeted therapies^[^^1^^]^.

DDR1 is a tyrosine kinase receptor that is enhanced by several collagens and regulates several signaling pathways, matrix remodeling, cell proliferation, and migration. DDR1 overexpression in invasive tumors confirms that the dysregulation of DDR1 enhances tumor progression^[^^30^^,^^31^^]^. Increased DDR1 expression is involved in the development and progression of NSCLC. Collagen type I elicits the upregulation of DDR1 expression in primary human lung fibroblasts at distinct concentrations and durations. In addition, collagen type I activates DDR1 pathway to promote epithelial-mesenchymal transition by the activation of MMP-9, which contributes to NSCLC cell invasion^[^^32^^,^^33^^]^. In the present study, we demonstrated that collagen type I could significantly increase the expression of DDR1 in human NSCLC cells. Therefore, DDR1 is a crucial clinical molecular target for NSCLC.

The truncated form of DARPP-32, t-DARPP, is expressed in adult brain striatal cells and various cancers such as gastric, breast, esophageal, prostate, and colon cancers. The t-DARPP lacks the phosphorylation site (T34) responsible for inhibiting PP1, whereas it possesses a phosphorylation site (T39) that activates PKA^[^^22^^-^^24^^]^. Many studies have been conducted on the role of t-DARPP in different types of cancer. Overexpression of t-DARPP induces PKA activity and AKT (protein kinase B) phosphorylation; thus, the signaling pathway for the cell survival enhances emphasizing the new oncogenic functions of t-DARPP^[^^34^^]^. DARPP-32 and t-DARPP promote SCLC growth through increasing the Akt/Erk-mediated proliferation and antiapoptotic signaling. DARPP-32 isoforms are overexpressed in SCLC patient-derived tumor tissues but are undetectable in physiologically normal lung. Achaete-scute homolog 1 transcriptionally activates DARPP-32 isoforms in human SCLC cells^[^^35^^]^.

In addition, the overexpression of t-DARPP induces resistance to the antiproliferative effects of trastuzumab in HER2+ breast cancer cells. The t-DARPP expression promotes antiapoptotic function in cancer cells by upregulating Bcl2^[^^21^^,^^26^^,^^36^^]^. Phosphorylation of DARPP-32 at the T75 location results in its transformation into a PKA inhibitor. Conversely, phosphorylation at T39, the analogous site in t-DARPP, is essential for the development of trastuzumab resistance and the initiation of the PI3K/Akt pathway. High levels of DARPP-32 reverse the effects of t-DARPP on trastuzumab resistance and PKA activity^[^^37^^]^. The expression of DARPP-32 and t-DARPP proteins plays an important role in promoting angiogenesis by regulating the expression and secretion of ANGPT2 in gastric cancer cells^[^^20^^]^. On the other hand, DARPP-32 has been shown to be a new DDR1 binding partner in human breast cells whose protein expression reduces or eliminates in several breast cancer cell lines. Moreover, DARPP-32 re-expression inhibits migration in breast tumor cells through the mechanism of DDR1 and Thr-34 phosphorylation. Co-expression of both DDR1 and DARPP-32 in MDA-MB-231 cells also inhibits migration, supporting a critical role of the DDR1/ DARPP-32 complex in motility. The phosphorylation of Thr-34 is necessary for the ability of DARPP-32 to impair breast tumor cell migration^[^^12^^,^^38^^]^. Therefore, the t-DARPP that lacks the Thr-34 phosphorylation site and overexpresses in different cancer cells is a novel and more appropriate factor to be evaluated in lung cancer. In gastric cancer, it has been found that NF-κB transcriptionally upregulates *PPP1R1B* gene expression in vitro and in vivo models^[^^39^^]^. The transcription factor NF-kB has an element at -1008 to -996 upstream of the *PPP1R1B* promotor for binding and regulating DARPP-32 isoforms expression^[^^39^^]^. Moreover, DDR1 activation has been shown to induce NFκB pathways^[^^28^^]^. Therefore, it can be suggested that DDR1 affects the expression of DARPP-32 and t-DARPP through the regulation of NF-κB. In the current study, we investigated the effect of DDR1 on t-DARPP expression in lung adenocarcinoma. Our results demonstrated that DDR1 protein promotes t-DARPP expression in lung cancer cells.

## CONCLUSION

Our findings suggest that t-DARPP is upregulated in NSCLC and its overexpression can facilitate the induction of oncogenesis. Therefore, t-DARPP could serve as a promising molecular target in NSCLC with prognostic and therapeutic value. We also propose that collagen type I influences oncogenic aspects of NSCLC cells through increasing the expression of t-DARPP, which is achieved by the induction of DDR1 expression. Further studies are needed to investigate the biological mechanisms by which t-DARPP affects lung tumorigenesis.

## DECLARATIONS

### Acknowledgments

This study was carried out in the National Institute of Genetic Engineering and Biotechnology (NIGEB), Tehran, Iran. The authors wish to express their special thanks to all the scientific members and staff of Medical Biotechnology Department at the National Institute of Genetic Engineering and Biotechnology (NIGEB), Tehran, Iran for their support and assistance. No artificial intelligence (AI)-assisted technologies was used in the production of this study.

### Ethical approval

Not applicable.

### Consent to participate

Not applicable.

### Consent for publication

All authors reviewed the results and approved the final version of the manuscript.

### Authors’ contributions

ZD: conceptualization, methodology, data analysis, acquisition, analysis, and interpretation of data, performing experiments, drafting of the manuscript, writing, review, and editing; PR: validation, investigation, interpretation of data; TM: resources, data curation, technical and experimental support; MH: conceptualization, funding acquisition, project administration, writing, review, and editing.

### Data availability

 The datasets used and analyzed during the present study are available from the corresponding author on reasonable request.

### Competing interests

The authors declare that they have no competing interests. 

### Funding

This study was financially supported by grant no. 787 of the National Institute of Genetic Engineering and Biotechnology (NIGEB), Tehran, Iran.

### Supplementary information

The online version does not contain supplementary material.
